# Correlation between herpes simplex virus neutralizing antibody titers determined by ELVIS cell and traditional plaque reduction assays

**DOI:** 10.1371/journal.pone.0214467

**Published:** 2019-04-04

**Authors:** Tamara P. Blevins, Yinyi Yu, Robert B. Belshe, Abbie R. Bellamy, Lynda A. Morrison

**Affiliations:** 1 Department of Medicine, Division of Infectious Diseases, Saint Louis University School of Medicine, St. Louis, Missouri, United States of America; 2 The Emmes Corporation, Rockville, Maryland, United States of America; 3 Department of Molecular Microbiology and Immunology, Saint Louis University School of Medicine, St. Louis, Missouri, United States of America; Cincinnati Children’s Hospital Medical Center, UNITED STATES

## Abstract

Preventive viral vaccine efficacy trials require large-scale sample analysis to quantitate immune responses and their correlation with infection outcomes. Traditional plaque reduction assays measure a functionally important form of humoral immunity, neutralizing antibody titer. These assays, however, are time-consuming and laborious. We previously developed a higher throughput assay of neutralizing antibody to herpes simplex viruses 1 and 2 (Blevins et al., PLOS ONE, 10(12), e0144738) using the enzyme-linked virus inducible system (ELVIS) cell line; this cell line produces β-galactosidase in response to HSV infection. Here, serum samples from recipients of an investigational vaccine in the Herpevac Trial for Women were used to compare the ELVIS cell assay with the lower throughput, traditional plaque reduction assay. We demonstrate that neutralizing antibody titers to HSV-1 or HSV-2 determined using ELVIS cells positively correlate with neutralizing antibody titers determined by traditional plaque reduction assay, thus validating a higher throughput alternative for large-scale sample analysis.

## Introduction

HSV enters cells via the interaction of glycoprotein D (gD) with specific cell surface receptors HVEM, nectin-1 and/or nectin-2 [1]. gH/gL and gB also participate in viral entry and fusion [2]. Natural viral infection or vaccination can generate antibodies that bind these important glycoproteins and, depending on the epitope recognized, interfere with their entry functions. Assays of antibody binding such as ELISA differ from neutralization assays in that the latter measure antibodies that block virus infectivity either at the stage of receptor binding, essential protein conformational changes, or interaction between proteins involved in fusion events at the plasma membrane or in the endosome. Thus, neutralizing antibodies represent a subset, indeed a functional subset, of all antiviral antibodies stimulated by infection or vaccination. Several types of assay for serum neutralizing antibodies have been developed. Traditional plaque reduction assays require mixing small quantities of virus with dilutions of serum plus complement, followed by addition to a cell monolayer. Plaque formation is then enumerated in a labor-intensive process. Modern higher throughput assays have been developed that utilize either beta-galactosidase (β-gal)-expressing virus [3,4] or an infection-responsive cell line [5–7], with the read-out of color intensity monitored by spectrophotometer. The latter assay utilizing ELVIS (Enzyme Linked Virus Inducible System) cells [8] provides a consistent, objective colorimetric readout that delivers reliable results within as little as 46 h. It has also been optimized for use with any HSV-1 or HSV-2 strain or clinical isolate [7], providing an advantage over the assays using a single virus engineered to express beta-galactosidase. A 50% cytopathic effect inhibition assay was previously compared with the original ELVIS microneutralization assay [5]. However, the ELVIS assay has not been compared with a traditional plaque reduction assay, and the extent to which the traditional and optimized higher throughput methods correlate is an important question for standardization of results between laboratories and between results from different vaccine trials.

The Herpevac Trial for Women included more than 8,000 initially HSV-1/2 seronegative women in a randomized, double-blind phase 3 trial of HSV-2 gD subunit vaccine in adjuvant. Results indicated no protection against subsequent HSV-2 infection, but 35% vaccine efficacy against HSV-1 infection and 58% vaccine efficacy against HSV-1 disease [9]. Serum antibody binding titers in gD-2 vaccine recipients, measured by ELISA, correlated with protection [10]. The extent to which neutralizing antibodies correlate with protection remains to be determined, and with thousands of sera stored from multiple study visits, a higher throughput method of analysis would be desirable. We therefore sought to determine how well HSV-specific serum neutralizing antibody titers correlate when determined by the ELVIS assay versus traditional plaque reduction assay to validate use of the new higher throughput assay.

## Methods

### Materials and methods

#### Sera and reagents

The positive control consisted of pooled normal human serum (Sigma Aldrich, St. Louis, MO) and a negative control consisted of human serum from an HSV-1/2 seronegative donor. Test sera were banked from the Herpevac Trial for Women [4] and included samples collected prior to vaccination (Month 0) and one month following the final vaccination (Month 7) from individual subjects who received three vaccinations and were not infected with HSV-1/2 during the trial. All study subjects were seronegative for HSV-1 and HSV-2 prior to entry into the clinical trial. The study was approved by the Saint Louis University Institutional Review Board (IRB number 24706; NCT00057330) and subjects provided written consent to future use of their samples. Collection of serum from an HSV-1/2 seronegative individual as an anonymous donation was approved by the Saint Louis University Institutional Review Board (IRB number 26646).

Lyophilized guinea pig serum with defined complement activity was obtained from Quidel Corporation (San Diego, CA).

#### Cells and viruses

Baby hamster kidney (BHK) cells stably transformed with a construct containing the HSV-1 ICP6 promoter driving the *E*. coli *lacZ* gene (ELVIS cells) [8], were obtained from Quidel, and were cultured as previously described [7]. Vero cells (African green monkey kidney epithelium; originally obtained from David Knipe) were propagated in Dulbecco’s MEM supplemented with 3% bovine growth serum, 3% newborn calf serum, 2 mM L-glutamine, and 100 IU/ml P/S.

The HSV-1 strain 5 and the HSV-2 strain 16 were grown in Vero cells from swab samples collected from infected subjects in the Herpevac Trial. Cell lysate stocks were prepared as previously described [7].

#### ELVIS neutralization assays

Month 0 and Month 7 sera from the Herpevac Trial were heat inactivated for 30 min at 56°C, then serially diluted in two-fold steps in a 96-well U-bottom plate (Corning) from 1:20 to 1:10,240 in 100 μl of EMEM supplemented with 2% FBS and 10% guinea pig serum as a source of complement. The diluted sera were used for simultaneous ELVIS and plaque reduction neutralization (PRNT) assays. Test serum dilutions and HSV-1 or HSV-2 were combined and incubated as previously described [7] before adding them directly onto each cell monolayer. Virus control wells (no human serum) and uninfected control wells (no virus) were included for each sample on the plate as well as a positive and negative serum control. After the virus/serum addition, the plates were incubated for 1 h and then EMEM containing 2% FBS was added and the plates were incubated overnight (18 h). The following day, the medium was carefully aspirated, and cell monolayers were lysed, frozen and developed as previously described [7].

IC_50_ values were calculated using a 4-parameter logistic dose response curve (GraphPad Prism). Briefly, the OD_570_ was plotted against the log serum dilution to generate a sigmoidal curve. Positive (no human serum) and negative (no virus) controls were used to define the constraints for the top and bottom of the curve, respectively, and the 50% neutralization titer was determined mathematically.

#### PRNT assay

Vero cells in 150 cm^2^ flasks were seeded into 24-well tissue culture-treated plates and incubated overnight to achieve confluent monolayers. The Month 0 and Month 7 sera were serially diluted and combined with HSV-1 or HSV-2 as described above. Mixtures were added in quantities to yield 50 to 100 plaques/well in the virus control wells. In addition to a negative and positive serum control, uninfected control wells (no virus) were included as an assay control. The virus/serum mixtures were incubated for 1 h at 37°C and 5% CO_2_ with gentle rocking. Following the incubation, 20 μl of serum dilutions 1:20 to 1:1,280 were added to Vero cell monolayers containing 130 μl of EMEM supplemented with 2% FBS and 10% guinea pig serum as a source of complement. After the virus/serum addition, the plates were incubated at 37°C, 5% CO_2_ for 1 h. Vero monolayers were washed with 1x PBS followed by an addition of 1 ml/well of DME containing 2% newborn calf serum and 0.5% normal human serum. The plates were incubated for 2 d at 37°C and 5% CO_2_. After the incubation, the medium was carefully aspirated, and the tissue culture monolayers were fixed with methanol for 10 min at room temperature. After the incubation, the methanol was decanted, tissue culture monolayers were stained with Giemsa overnight at room temperature. The following day, the Giemsa stain was aspirated from the plates, the monolayers were washed with water, and the plates were air dried.

Plaques were counted using a dissecting microscope, and the endpoint serum neutralizing antibody titer of each serum sample was defined as the reciprocal of the highest dilution of the serum with a mean plaque count less than or equal to the 50% plaque reduction cutoff value for the assay. Plaque counts above and below the 50% plaque reduction endpoint (assay cutoff) were entered into an Excel file where the endpoint titer was interpolated by linear regression.

### Statistical methods

The primary objective of this analysis was to compare the neutralizing antibody titers obtained by the ELVIS assay with those obtained by the plaque reduction assay using samples from the Herpevac Trial available for future use. Sample sizes for the evaluation were determined experientially based on previous assessments of similar types; the sample size was not designed to test a formal null hypothesis. Titers were summarized with geometric means and corresponding 95% confidence intervals and compared using Spearman correlation and linear regression. Tests were 2-sided with a significance threshold of α = 0.05. All analyses were conducted using SAS v9.4 (SAS Institute Inc.).

## Results

To establish basic parameters for comparison of ELVIS and plaque reduction neutralization assays, we used pooled human serum because it contains anti-HSV-1 and HSV-2 antibodies due to the prevalence of HSV infection in the general population and thus serves as a positive control serum for both viruses. In pooled human sera, the average titer derived from three independent assays of HSV-1 neutralizing capacity was 1:346 in ELVIS and 1:325 in traditional plaque reduction assays, and of HSV-2 was 1:132 in ELVIS and 1:108 in plaque reduction assays (Table 1). The largest discrepancy between ELVIS and traditional assay results in a single assay was 1.6-fold. Serum from a defined HSV-1/HSV-2 seronegative individual consistently registered no neutralizing antibody titer in both assays (defined as half the cutoff of 20).

**Table 1:** Titers of HSV-specific neutralizing antibody in positive control serum measured by ELVIS and traditional PRNT assays.

HSV-1/2 positive control serum				
	HSV-1		HSV-2	
Assay	ELVIS PRNT	Traditional PRNT	ELVIS PRNT	Traditional PRNT
1	436	437	92	120
2	295	290	159	101
3	307	248	146	103
Mean ± SEM	346 ± 45	325 ± 57	132 ± 21	108 ± 6

**Fig 1:**
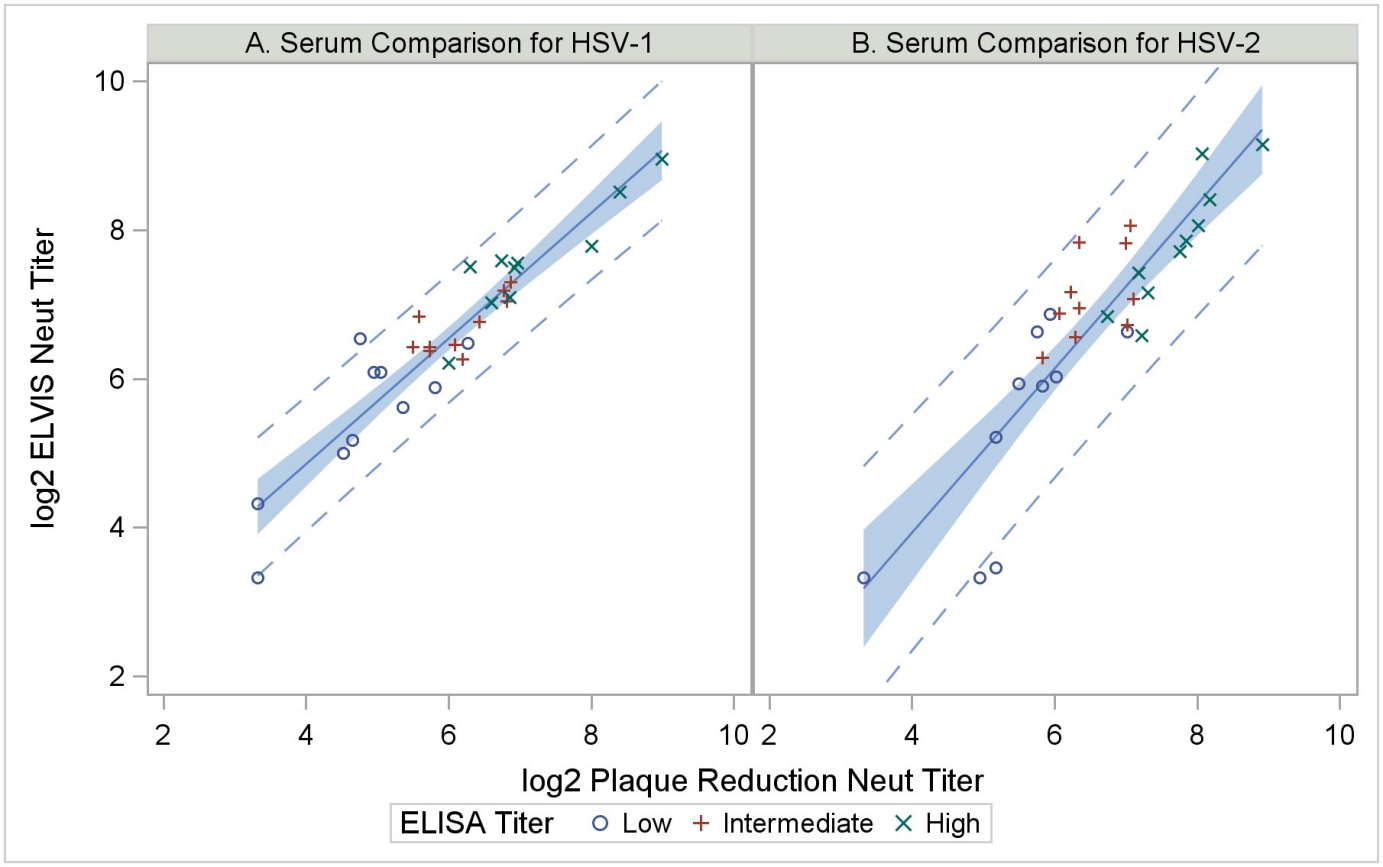
Correlation between anti-HSV titers of sera from women vaccinated with HSV-gD2 vaccine in ELVIS and traditional PRNT assays. Month 7 sera were diluted and tested simultaneously in ELVIS and traditional PRNT assays. Dots represent individual serum samples, with marker shapes indicating previously reported ELISA titer level [9]. Solid lines indicate predicted values from linear regression analysis with the shaded band showing the 95% confidence interval and the dashed lines showing the 95% prediction interval A) HSV-1 (R^2^ = 0.87); B) HSV-2 (R^2^ = 0.78).

We then applied the assay comparison to sera from the Herpevac Trial participants, analyzing Month 0 and Month 7 sera from 30 individuals whose ELISA titers had been designated as low (median: 2,870; range: 651–4,785), intermediate (median: 6,795; range: 5,297–9,543), or high (median: 13,516; range: 10,685–34,912). Demographic makeup of subjects included in the sample set was similar to the parent trial: women were on average 20 years old and the majority were white and non-Hispanic [9]. Neutralizing antibody results against HSV-1 and HSV-2 in the presence of complement were non-detectable in Month 0 samples in both ELVIS and plaque reduction assays. Geometric mean titers measured by each assay are presented by virus type in Table 2 for all subjects and stratified by ELISA titer (low/intermediate/high) as determined previously [9]. A strong and significant positive correlation existed between neutralizing antibody titers determined by ELVIS versus plaque reduction assays for both HSV-1 (Spearman correlation coefficient, r = 0.90, p <0.001) and HSV-2 (r = 0.85, p<0.001). This correlation was confirmed by linear regression of log transformed ELVIS titer against log-transformed PRNT titer (Fig 1).

**Table 2:** Geometric mean titers (95% confidence interval) of ELVIS and traditional PRNT and ELISA assays on Herpevac Trial sera in the presence of complement.

		ELISA Titer Sampling Group			
Virus	Assay	Low (N = 10)	Intermediate (N = 10)	High (N = 10)	All (N = 30)
HSV-1	Neut—ELVIS	44 (26–73)	105 (87–126)	190 (130–278)	96 (71–129)
	Neut—PRNT	28 (17–45)	72 (55–94)	145 (90–234)	66 (48–92)
HSV-2	Neut—ELVIS	40 (20–82)	141 (105–189)	226 (147–347)	109 (75–158)
	Neut—PRNT	44 (28–71)	92 (73–117)	211 (155–289)	95 (71–129)
HSV-1/2	ELISA	2513 (1628–3877)	7162 (6267–8186)	15745 (11667–21248)	6568 (4739–9103)

**Fig 2:**
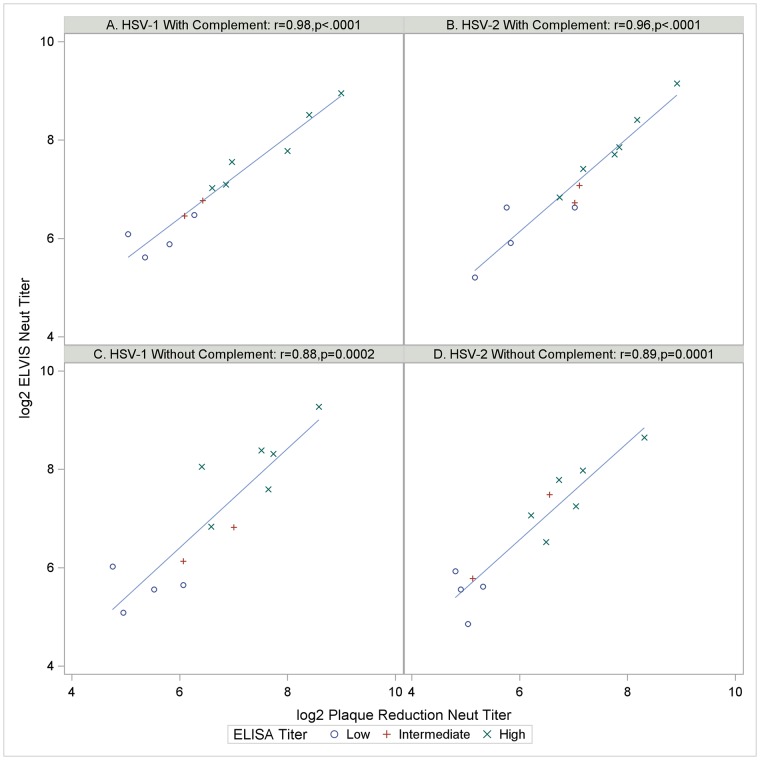
Correlation between anti-HSV titers of sera in ELVIS and traditional PRNT assays in the presence and absence of added complement. Nine month 7 sera were diluted and tested simultaneously in ELVIS and traditional PRNT assays. Dots represent individual serum samples, with marker shapes indicating previously reported ELISA titer level [9]. Solid lines indicate predicted values from linear regression analysis with the shaded band showing the 95% confidence interval and the dashed lines showing the 95% prediction interval. A) Anti-HSV-1 or B) anti-HSV-2 with complement; C) anti-HSV-1 or D) and-HSV-2 without added complement.

Lastly, we assessed the impact of complement addition on the correlation between titers obtained by ELVIS and PRNT assays using a subset of 12 sera used in Fig 1. A strong and significant positive correlation existed in the presence of complement between neutralizing antibody titers determined by ELVIS versus plaque reduction assays (Fig 2) for both HSV-1 (Spearman correlation coefficient, r = 0.98, p <0.0001) and HSV-2 (r = 0.96, p <0.0001). The correlation weakened slightly in the absence of complement for both HSV-1 (r = 0.88, p = 0.0002) and HSV-2 (r = 089, p = 0.0001). Neutralization of HSV-1 was 1.5-fold greater than of HSV-2 in the absence of complement, and no difference between the two viruses was observed in the presence of complement.

## Discussion

Our results using Herpevac Trial sera clearly demonstrate that neutralizing antibody titers determined by the higher throughput ELVIS cell assay closely correlate with titers determined by plaque reduction neutralization assay for anti-HSV-1 and anti-HSV-2 activity. Addition of complement enhances the correlation between the two types of assay. The correlation was revealed despite the markedly different read-outs of the ELVIS assay versus traditional plaque assay (OD units resulting from stimulation of β-gal expression upon infection versus plaques as a measure of infectious virus). These comparisons engender confidence that the higher throughput ELVIS cell assay can be used to derive an accurate depiction of neutralizing antibody titers while also being scalable for extensive sample analyses inherent in phase 2 and phase 3 vaccine trials.

We also observed that stratification of sera by ELISA titer (low/medium/high) [9] generally corresponds to the magnitude of their respective neutralizing antibody titers. Interestingly, neutralizing antibody titers are slightly higher in the ELVIS assay of HSV-1 neutralizing capacity than in the traditional plaque reduction assay. In contrast, the traditional neutralizing assay yields slightly higher titers against HSV-2 for normal human serum and certain Herpevac Trial sera than does the ELVIS cell assay. It is possible that the promoter in ELVIS cells is somewhat differentially responsive to HSV-1 versus HSV-2, such that any HSV-2 not neutralized by antibody would drive β-gal expression more forcefully than HSV-1, resulting in lower apparent antibody titers against HSV-2.

Addition of complement to the higher throughput ELVIS assay may explain the higher neutralizing antibody titers reported in this and a previous study [7] of Herpevac Trial sera compared with titers reported for the same sera by Awasthi et al. [11] and Belshe et al. [9]. Awasthi et al. also had detected 3.5-fold greater neutralization of HSV-1 than HSV-2 by sera from gD-vaccinated subjects using a plaque reduction assay in the absence of complement. Interestingly, when they analyzed neutralizing antibody responses to primary rather than laboratory virus isolates, the differential between neutralization of HSV-1 and HSV-2 strains dropped to 2.3-fold [11], suggesting virus strain-dependent differences may also have influenced the differential in neutralization of HSV-1 versus HSV-2 they had observed. Only slightly (1.5-fold) greater neutralization of HSV-1 than HSV-2 occurs in our assays in the absence of complement, and no difference between the two viruses is observed when complement is added [7]. Arguably, addition of complement best reproduces the neutralizing environment *in vivo*.

Our optimized and validated assay is higher throughput than the traditional plaque reduction assay and so will be useful in large-scale assessment of neutralizing antibody titers. It can be performed in a minimum of 46 h (from seeding cells in the late afternoon of day 1 to data acquisition in the early afternoon of day 3). Only about 3 h of technician time is required for sample and plate manipulation, depending on the number of plates prepared at one time, and up to 10 plates can easily be manipulated per assay. Four serum samples can be run per plate against a single virus isolate, so up to 20 sera can be tested against both HSV-1 and HSV-2 when using 10 plates per assay [7]. By staggering assays, as many as 3 may be completed during a 5 d work week. Thus, in all anti-HSV-1 and HSV-2 neutralizing antibody titers of up to 60 sera can be readily determined by a single technician in the course of a 5 d week. In contrast, anti-HSV-1 and HSV-2 neutralizing antibody titers of only 11 sera can be tested per 13-plate traditional plaque reduction assay, for a total of 33 sera over 3 assays per 5 d week. Plaque counting adds approximately 2 h more time per assay or 6 labor-intensive hours per 5 d week. Lastly, the ELVIS assay allows testing of serum dilutions from 1:20 to 1:10,240, but only dilution factors of 1:20 to 1:640 may be tested in the PRNT assay given the parameters described above. The 2-fold increase in throughput of the ELVIS assay compared with plaque reduction assay has led to a significant time savings in our measurement of neutralizing antibody titers of many thousands of samples from the Herpevac Trial currently being conducted.

The vaccine used in the Herpevac Trial consisted of the ectodomain of gD-2 and therefore focused the mechanisms of neutralization exclusively on gD. Neutralizing activity could involve blockade of gD-receptor interactions, or interaction of gD with gH/gL [12]. Regions of gD that participate in interactions with cellular receptors HVEM and nectin-1 have been identified through solution of the crystal structures when bound [13,14]. The gD–gH/gL interaction facilitates fusion [15], and several lines of evidence suggest the C-terminal portion of gD is involved [15–18]. Antibodies to two discontinuous epitopes on gD block gD interaction with gH/gL, one of which is HSV-2-specific and maps to the C-terminal portion of gD [15]. In addition to these epitopes, three antibody epitopes have been mapped that disrupt gD interaction with one or more of its cellular receptors [15]. Of these five epitopes, the gD ectodomain used in the vaccine elicits populations of antibodies that can block gD-gH/gL interactions (both discontinuous epitopes), or gD-receptor interactions (2 of 3 epitopes) [12]. The strongest correlate of neutralizing activity is capacity to recognize several of the five epitopes [12]. The ELVIS cell-based assay, which relies on whole infectious virus, would reflect activities of the same constellation of antibody populations recognizing epitopes in gD-gH/gL and gD-cellular receptor groups as the traditional plaque reduction neutralization assay.
